# Supplementary material for: Development and multi-site external validation of a generalizable risk prediction model for bipolar disorder
Source: Transl Psychiatry. 2024 Jan 25;14:58. doi: 10.1038/s41398-023-02720-y (PMC10810911; doi:10.1038/s41398-023-02720-y)
Supplement: Supplementary file 1 — Supplemental Material [file 41398_2023_2720_MOESM1_ESM.docx]

**Supplemental Material**

*Table S1: Clinical variables considered in developing predictive models for Bipolar Disorder*

| *Feature Category* | *# of Variables (VUMC)* | *# of Variables (MGB)* | *# of Variables (GHS)* | *Details* |
| --- | --- | --- | --- | --- |
| *Demographics* | *3* | *3* | *3* | *Age, Sex, Race* |
| *Comorbidities* | *135* | *130* | *130* | *All comorbidities are mapped to CCS code* |
| *Medications* | *2367* | *1712* | *1134* | *All medications are mapped to RxNorm ingredient* |
